# Prevalence of and Factors Associated With Dental Health Service Utilization in Mexican Pediatric Patients Aged 2–12 Years With High Dental Caries Experience

**DOI:** 10.7759/cureus.91032

**Published:** 2025-08-26

**Authors:** Salvador E Lucas-Rincón, Sandra I Jimenez-Gayosso, Norma-Leticia Robles-Bermeo, Rogelio J Scougall-Vilchis, Martha Mendoza-Rodríguez, Juan A Casanova-Sarmiento, Gladys R Acuña-González, América P Pontigo-Loyola, Mariana Mora-Acosta, Mauricio Escoffié-Ramírez

**Affiliations:** 1 Academic Area of Dentistry of Health Sciences Institute, Autonomous University of Hidalgo State, Pachuca, MEX; 2 Advanced Studies and Research Center in Dentistry “Dr. Keisaburo Miyata” School of Dentistry, Autonomous University of Mexico State, Toluca, MEX; 3 School of Dentistry, Autonomous University of Campeche, Campeche, MEX; 4 School of Dentistry, Autonomous University of Yucatan, Yucatán, MEX

**Keywords:** dental caries, dental health services, mexico, oral health, oral hygiene

## Abstract

Background

Oral health in children is essential for their overall development, but access to dental services remains unequal, especially among vulnerable populations. This study aimed to assess the prevalence and associated factors of dental health service utilization in Mexican pediatric patients with high dental caries experience aged 2-12 years visiting a university clinic in Toluca, Mexico.

Methodology

This retrospective, cross-sectional study analyzed 309 medical records of pediatric patients from the Pediatric Dentistry Clinic of the Autonomous University of the State of Mexico (UAEM). Data were extracted from records completed by parents/guardians, including variables such as age, sex, oral hygiene habits, number of siblings, caries experience (measured via WHO-standardized decayed, missing, and filled primary teeth (dmft) and decayed, missing, filled permanent teeth (DMFT) indices for primary and permanent dentition), previous dental pain experience, and current reason for consultation. Statistical analysis was performed using Stata version 14.0. Descriptive statistics (means, frequencies) characterized the sample, while bivariate analyses (chi-square, Mann-Whitney U tests) explored associations between variables. A binary logistic regression model identified predictors of dental health services utilization, adjusting for covariates. Significance was set at p-values ≤0.05.

Results

The mean age was 5.71 ± 2.43 years, and 50.8% were male. Of the children, 49.2% had previous dental visits. It was observed that for each year of age, the likelihood of having a previous dental visit increased by 44% (1.29-1.62). On the other hand, when the combined dmft + DMFT index increased by one unit, the odds of having a previous dental visit increased by 1.07 times (1.01-1.14).

Conclusions

Dental health services utilization in this sample of Mexican children with high dental caries experience was driven primarily by age and the presence of caries, reflecting late access and predominantly curative treatment. These results underscore the urgency of strategies that promote preventive care from early childhood, with an emphasis on vulnerable populations.

## Introduction

Oral health in the pediatric population is a fundamental component of overall well-being, with significant implications for children’s physical, social, and emotional development [1,2]. A child’s smile does more than reflect happiness; it serves as a window into their general health, educational potential, and future socioeconomic opportunities. Oral diseases, such as dental caries, represent one of the most prevalent public health problems in this age group, affecting not only oral functionality but also quality of life [3,4]. Dental caries in schoolchildren represents a global public health crisis with profound implications.

The 2021 Global Burden of Disease data revealed that oral diseases remain a massive public health challenge, affecting nearly 3.7 billion people worldwide, with untreated dental caries in permanent teeth and severe periodontitis being the most prevalent conditions. While the overall global age-standardized rates have seen relatively minimal change since 1990, the burden is shifting geographically; the African and Eastern Mediterranean regions are experiencing the largest increases in both prevalent cases and disability-adjusted life years (DALYs) for most conditions, whereas the European region is the only one showing a decline in the prevalence of untreated caries in both deciduous and permanent teeth. Notably, edentulism, severe periodontitis, and oral cancer cause the highest health burden in terms of disability, and despite stable global rates, the sheer number of affected individuals is growing for all conditions, except untreated deciduous caries, which saw no change, and orofacial clefts, which stand as a major success story with a dramatic 68.3% decrease in DALYs due to improved care [5]. Despite advancements in prevention and treatment, access to and utilization of dental services for children remain unequal, especially in socioeconomically disadvantaged contexts [6-8]. This inequality creates what researchers term the “oral health paradox,” those who need care most often receive it least [9].

The utilization of dental health services is shaped by a complex interplay of behavioral, sociodemographic, socioeconomic, and clinical factors that operate across multiple levels. Previous studies have highlighted that predisposing, enabling, and need factors are associated with dental health service utilization. According to the Andersen and Newman model [10], predisposing factors refer to individual characteristics influencing the likelihood of a person’s seeking dental care, such as parental education, oral hygiene habits, and previous dental experiences. Recent evidence suggests that these factors are particularly potent in pediatric care; children whose parents had negative dental experiences are more likely to delay their first dental visit [11]. Enabling factors represent the structural determinants that facilitate or hinder care access. These include socioeconomic status (households in the lowest income quartile have fewer preventive visits than higher-income groups [12]), geographic access to dental services (with rural populations facing average travel times longer than urban counterparts), and health insurance coverage. In Mexico’s fragmented health system, this is clearly evident; children covered by public health insurance are more likely to receive preventive/curative dental care or incur no out-of-pocket costs than those without insurance, despite having identical clinical needs [13-16]. Need factors reflect the clinical urgency driving care-seeking behavior, encompassing oral health conditions from dental caries to periodontal disease. The pain-discomfort axis proves particularly influential. Most pediatric dental visits in resource-limited settings occur only after symptom onset [17], often when irreversible damage has already occurred. However, critical gaps persist in understanding how these variables interact in pediatric populations, especially where health systems face triple burdens of poverty, malnutrition, and dental workforce shortages [2,3].

In Mexico, the burden of oral diseases in children is high, with a significant prevalence and incidence of caries in both primary and permanent dentitions [18-24]. The situation represents a perfect storm of dietary changes, inadequate prevention programs, and systemic barriers to care. For example, the National Caries and Dental Fluorosis Survey [25] and the National School Health Survey [26] reveal that approximately 50% of school-aged children have or report caries experience, underscoring the need for effective strategies to promote the utilization of both preventive and curative dental health services. Likewise, dental care for children generally focuses on treating pain or infections rather than adopting a more preventive approach. This situation is more pronounced in environments where specialized services are scarce or difficult to access, such as rural or marginalized urban areas [3]. Additionally, the literature indicates that past negative experiences at the dentist can lead to dental anxiety and avoidance of future visits, perpetuating a cycle of disease and delayed treatment [27-29].

On the other hand, practices such as parent-assisted oral hygiene and regular toothbrushing frequency are associated with a lower prevalence of caries and a greater predisposition to preventive care [3,30,31]. Successful programs have demonstrated that school-based brushing initiatives can reduce caries incidence in high-risk populations [32-34]. However, studies analyzing how these variables interact with dental service utilization in children, particularly in Latin American contexts, remain limited. This study provides significant evidence regarding research on dental service utilization in Mexican children with a high prevalence of dental caries, an underexplored public health problem in resource-limited settings. The findings, supported by a meticulous multivariate analysis, highlight a critical reality: the system prioritizes treatment over prevention. Furthermore, it provides scientific grounds for restructuring public policies, underscoring the urgent need to implement scalable preventive programs in vulnerable settings. Based on the Andersen-Newman behavioral model and the documented socioeconomic barriers in the region, we hypothesized that the utilization of dental health services in Mexican pediatric patients would be significantly associated with clinical need factors (older age and higher caries experience) rather than predisposing (e.g., sex, oral hygiene habits) or enabling factors (e.g., family size). The objective of this study was to assess the prevalence and factors associated with prior dental service use among children aged 2-12 years visiting a university clinic in Toluca, Mexico.

## Materials and methods

Study population and sample

This cross-sectional study was conducted among children aged 2-12 years. Participants were selected through non-probabilistic sampling. To calculate the sample size, a 95% confidence level, a 4% margin of error, an estimated prevalence of 90%, and an expected loss rate of 10% were considered, resulting in a total of 314 individuals. Part of the methodology has been previously published for other health indicators [19,35,36].

Inclusion criteria for medical records were patients of both sexes, records with complete diagnostic data, ages between two and 12 years, clinic authorization for treatment, and signed informed consent. Exclusion criteria were those for which parents did not permit clinical photographs to be taken.

The final sample consisted of 309 patient records from the Pediatric Dentistry Clinic of the Autonomous University of the State of Mexico (UAEM), located in Toluca de Lerdo. This public institution offers low-cost specialized dental services, primarily catering to low- and middle-income populations while being open to individuals from all socioeconomic levels in the region.

Data collection and variables

Initially, authorization was obtained from the Program Coordination. All data were collected from medical records previously completed by the patients’ parents and/or guardians. The dependent variable was the previous utilization of dental health services (before the current consultation, before contacting the university clinic), coded as 0 = no and 1 = yes.

Independent variables included in the study were (1) patient age (range = 2-12 years); (2) sex (coded: 0 = male, 1 = female); (3) assisted oral hygiene (0 = no, 1 = yes); (4) toothbrushing frequency (times per day, reported by the guardian); (5) number of siblings (reported by the guardian); (6) dental caries experience (evaluated in each dentition type); (7) previous dental pain experience (0 = never, 1 = yes, previously, 2 = yes, currently); and (8) current reason for consultation (0 = pain, 1 = check-up, 2 = rehabilitation, 3 = other).

Caries assessment

The dmft index, used to assess dental caries in the primary dentition, considers the number of decayed, extracted, or indicating extraction, and filled teeth. The “m” component (missing) was included only for teeth that were explicitly documented in the clinical record as having been extracted due to caries. Teeth absent due to physiological exfoliation or with an unknown reason for absence were not counted. On the other hand, the DMFT index, used to assess dental caries in the permanent dentition, considers the number of decayed, missing, and filled teeth. These indices were calculated according to the criteria established by the WHO to quantify the current and historical prevalence of caries [37]. These indices were obtained from diagnoses recorded in medical records by pediatric dentistry students under the close supervision of experienced instructors, and subsequently, validated through the analysis of clinical photographs.

A global caries index, dmft + DMFT, was constructed because there were children with only primary dentition and children with only permanent dentition; the sample size could be reduced, and we would not capture the effect of caries experience on oral health service utilization. On the other hand, the study did not aim to identify factors associated with caries experience; for this, separate analyses would be required. The combined index assesses the total burden of caries experience in populations with transitional dentition.

Statistical analysis

Data processing was performed using Stata software version 14.0. The analysis in this study included the below.

Univariate Analysis

For qualitative variables, absolute and relative frequencies (percentages) were calculated. For quantitative variables, measures of central tendency and dispersion (means and standard deviations) were determined.

Bivariate Analysis

To evaluate differences in previous dental service utilization based on the studied indicators, the statistical tests used were the chi-square test (χ²) for categorical variables and the Mann-Whitney U test for ordinal or quantitative variables vs. a qualitative variable with two categories.

Multivariate Analysis

For multivariate analysis, a binary logistic regression model was used, where having used dental service served as the dependent variable and was controlled for the presence of confounding factors. The variable selection method used an evidence-based strategy, where only candidate covariates that showed an initial association in bivariate analyses (p < 0.25) were included. This conservative threshold helped retain relevant variables that might achieve significance in adjusted models. Model calibration between predicted and observed probabilities was realized with the Hosmer-Lemeshow goodness-of-fit test. Before final model construction, we assessed multicollinearity among predictors using variance inflation factors (VIFs), with values ​​exceeding 5.0 indicating problematic correlation requiring variable exclusion. An alpha level of 0.05 was used for statistical significance; odds ratios (ORs) and 95% confidence intervals (CIs) were used to measure effect size. Continuous variables (e.g., age, dmft + DMFT index) were modeled per unit increase to enhance clinical interpretability [38]. The final results were organized in adherence to the Strengthening the Reporting of Observational Studies in Epidemiology (STROBE) statement.

Ethical considerations

This study complied with the guidelines established in the General Health Law on Research [39]. As only a review of clinical records was conducted, obtaining informed consent from the patients was not required. The research protocol was officially approved by the Faculty of Dentistry of the Autonomous University of the State of Mexico (CIEAO/16/05). Additionally, a secondary analysis of the data was reviewed and approved by the Research Ethics Committee of the Stomatology Research Network (CEIRIE-001.25), which further validated the study’s ethical and methodological rigor.

## Results

A total of 309 medical records of participants attending the pediatric dentistry clinic were reviewed. Table 1 presents the general characteristics of the sample. The mean age was 5.71 ± 2.43 years, the mean number of siblings was 1.37 ± 1.03, and the mean daily toothbrushing frequency was 2.71 ± 0.71 times. The mean combined dmft + DMFT index was 9.10 ± 4.19. The distribution by sex was also presented (50.8% male), as well as previous dental pain experience (56.6% with a history of pain), assisted oral hygiene (50.5% without assistance), and reason for consultation (38.5% for rehabilitation). Prevalence of previous dental visits, our dependent variable, was 49.2%, with 50.8% of children without previous visits.

**Table 1:** Descriptive analysis of the variables included in the study.

Variable	Mean (median)	SD
Age	5.71 (5.24)	2.43
Number of siblings	1.37 (1.00)	1.03
Daily toothbrushing frequency	2.71 (3.00)	0.71
dmft + DMFT index	9.10 (9.00)	4.19
	Frequency	Percentage
Sex		
Male	157	50.8
Female	152	49.2
Previous dental pain experience		
Never	175	56.6
Yes, previously	95	30.7
Yes, currently	39	12.6
Assisted oral hygiene		
No	156	50.5
Yes	153	49.5
Current reason for consultation		
Pain	68	22.0
Check-up	62	20.1
Rehabilitation	119	38.05
Other	60	19.4
Previous dental visits (dependent variable)		
No	157	50.8
Yes	152	49.2

Table 2 compares the characteristics of patients based on their history of previous dental visits (no visits vs. previous visits), using non-parametric tests. Significant differences were found in age (p = 0.0000), analyzed using the Mann-Whitney U test. However, no significant differences were observed for the number of siblings, daily toothbrushing frequency, or the combined dmft + DMFT index. Additionally, a significant association was found with assisted oral hygiene (p = 0.005, chi-square test), whereas no statistically significant differences were observed for variables such as sex, dental pain experience, or reason for consultation.

**Table 2:** Bivariate analysis between previous dental visits and independent variables included in the study. ^*^: Mann-Whitney; ^†^: chi-square.

Variable	No visit	With visits	P-value
Age	4.81 ± 2.17	6.63 ± 2.35	<0.0001^*^
Number of siblings	1.26 ± 0.95	1.48 ± 1.10	0.1404^*^
Daily toothbrushing frequency	2.66 ± 0.77	2.77 ± 0.65	0.1624^*^
dmft + DMFT Index	8.76 ± 3.99	9.46 ± 4.37	0.3625^*^
Sex			
Male	78 (49.7)	79 (50.3)	0.687^†^
Female	79 (52.0)	73 (48.0)	
Previous dental pain experience			
Never	94 (53.7)	81 (46.3)	0.341^†^
Yes, previously	47 (49.5)	48 (50.5)	
Yes, currently	16 (41.0)	23 (59.0)	
Assisted oral hygiene			
No	67 (42.9)	89 (57.1)	0.005^†^
Yes	90 (58.8)	63 (41.2)	
Current reason for consultation			
Pain	29 (42.6)	39 (57.4)	0.207^†^
Check-up	38 (61.3)	24 (38.7)	
Rehabilitation	60 (50.4)	59 (49.6)	
Other	30 (50.0)	30 (50.0)	

Table 3 shows the results of the multivariate analysis using binary logistic regression. It was found that for every additional year of age, the likelihood of having a previous dental visit increased by 44% (1.29-1.62). On the other hand, for each unit increase in the combined dmft + DMFT index, the odds of having a previous dental visit increased by 1.07 times (1.01-1.14). Model calibration between predicted and observed probabilities was adequate (Hosmer-Lemeshow goodness-of-fit test; chi-square = 11.25; p = 0.188).

**Table 3:** Multivariate analysis between previous dental visits and independent variables included in the final model. *: Reference category. Goodness-of-fit test: Hosmer-Lemeshow chi2(8) = 11.25; p = 0.1880. OR: odds ratio; CI: confidence interval

Variable	OR (95% CI) crude	OR (95% CI) adjusted	P-value
Sex			
Male	1*		
Female	0.91 (0.58 – 1.42)	0.94 (0.57 – 1.53)	0.815
Age	1.40 (1.36 – 1.56)	1.44 (1.29 – 1.62)	0.000
dmft + DMFT index	1.04 (0.98 – 1.09)	1.07 (1.01 – 1.14)	0.014

## Discussion

The results of this study reveal that dental service utilization in Mexican children attending a university clinic is approximately 50% and is significantly associated with age and caries experience (dmft + DMFT index). This aligns with previous findings emphasizing the critical role of caregivers in children’s oral health [32]. The higher prevalence of dental visits in older children may be explained by the accumulation of treatment needs or by greater parental awareness of the importance of preventive care as children grow older [15,40,41]. In this regard, as children grow older, the prevalence of caries and oral complications increases, raising the likelihood of consultations for pain or rehabilitation [21]. Furthermore, caregivers may perceive dental care as more necessary for school-aged children than for preschoolers, particularly when visible issues or symptoms are present [32]. These findings provide relevant evidence for understanding dental care access patterns in vulnerable pediatric populations and highlight the need to strengthen preventive strategies from an early age.

The significant differences in the dmft + DMFT index between children with and without previous dental visits suggest that service utilization is primarily driven by existing oral health needs, which could translate into better oral health outcomes in the population, considering the high caries indices in this sample. This could be attributed to the fact that dental visits in this context are more focused on curative treatment than on prevention, as indicated by the high percentage of consultations for rehabilitation and pain. This pattern reflects a global trend in vulnerable populations, where access to preventive services is limited [3]. This finding supports the Andersen and Newman model [10], where “need factors” (e.g., evident disease) are key determinants of dental health service utilization. Nevertheless, it also reflects a pattern in dental care utilization similar to that documented in other contexts with limited access to preventive care [17].

The absence of significant associations between dental service utilization and variables such as sex, daily toothbrushing frequency, or family size (measured by number of siblings) reveals an important pattern in healthcare access dynamics. These findings align with emerging evidence suggesting that conventional predictors of health behavior become less influential when structural barriers dominate the care-seeking equation [17,42,43]. Specifically, when fundamental obstacles such as geographic clinic availability (particularly in rural areas), prohibitive treatment costs, or insurance coverage limitations are present, they appear to override the effects of individual-level characteristics [25,44,45]. This phenomenon has been documented in similar low-resource settings where systemic constraints create a “floor effect,” the structural barriers are so substantial that they minimize observable differences between demographic groups or behavioral patterns [17,20]. Our results particularly emphasize this in the Mexican context, where even children with better oral hygiene practices showed no greater likelihood of accessing care, suggesting that preventive behaviors alone cannot compensate for systemic access barriers. These insights underscore the need for policy interventions that address these foundational structural issues alongside traditional health promotion strategies.

This study has some limitations. First, the cross-sectional design prevents establishing causal relationships between the analyzed variables and dental service utilization. Second, the sample was limited to patients from a university clinic, which may affect the generalizability of the results to other contexts, especially in rural areas with less access to specialized services. Third, the data relied on clinical records and parental reports, introducing potential recall bias or underreporting. Finally, detailed socioeconomic variables, such as family income or parental education level, were not included. These variables could help in understanding inequalities in service utilization. Although the sample included only children who accessed the university clinic, the comparison between those with and without previous dental visits revealed that factors such as age and caries severity influence early service utilization, even within populations that eventually seek care. Future studies should evaluate barriers among children who have never received dental attention.

These results suggest that future research should address several challenges. First, longitudinal and self-report studies can help establish causal relationships. Second, future surveys should incorporate information from respondents about their socioeconomic status, including income and parental education. Third, future research should focus on preventive interventions in vulnerable communities. Fourth, more comprehensive data should be collected to detect structural barriers and explore other types of variables. Fifth, future research should also examine whether the dental model can be integrated with family medicine and, if so, how these approaches will contribute to developing effective policies for reducing childhood caries and equitable access to preventive dental services.

## Conclusions

Our findings demonstrate that dental service utilization among Mexican pediatric patients with high caries experience is primarily driven by age and caries experience (dmft + DMFT index), reflecting a reactive rather than preventive healthcare-seeking pattern. This trend underscores a systemic failure in which dental care is sought predominantly at advanced stages of disease progression, often triggered by pain or functional impairment. The strong association with caries severity and age highlights that caregivers prioritize treatment needs over preventive measures, likely due to a combination of limited oral health literacy, economic constraints, and structural barriers such as geographic access and cost. These results align with Andersen’s behavioral model, where “need factors” override predisposing and enabling characteristics in resource-limited settings. The absence of significant associations with variables such as toothbrushing frequency or sex further emphasizes that individual behaviors are overshadowed by broader structural inequities. The persistent pattern of late-stage, curative care demands urgent policy interventions and community-level strategies. Future efforts should focus on integrating oral health into primary care systems, implementing school-based preventive programs (e.g., fluoride varnish applications, sealants), and designing targeted educational campaigns for caregivers of young children to disrupt the cycle of neglect. Additionally, longitudinal studies are needed to explore causal pathways and evaluate the impact of decentralized dental care models (e.g., mobile clinics, tele-dentistry) on reducing barriers to early prevention. By shifting the focus from restorative to preventive care, healthcare systems can mitigate the long-term health and economic burdens of pediatric caries in vulnerable populations.

## References

[1] Han SY, Chang CL, Wang YL (2025). A narrative review on advancing pediatric oral health: comprehensive strategies for the prevention and management of dental challenges in children. Children (Basel).

[2] Peres MA, Macpherson LM, Weyant RJ (2019). Oral diseases: a global public health challenge. Lancet.

[3] National Institute of Dental and Craniofacial Research (2021). Effect of oral health on the community, overall well-being, and the economy. Oral Health in America: Advances and Challenges.

[4] Bukhari OM (2020). Dental caries experience and oral health related quality of life in working adults. Saudi Dent J.

[5] (2025). Trends in the global, regional, and national burden of oral conditions from 1990 to 2021: a systematic analysis for the Global Burden of Disease Study 2021. Lancet.

[6] World Health Organization (2023). Global Oral Health Status Report: Towards Universal Health Coverage for Oral Health by 2030. World Health Organization.

[7] Almajed OS, Aljouie AA, Alharbi MS, Alsulaimi LM (2024). The impact of socioeconomic factors on pediatric oral health: a review. Cureus.

[8] Verlinden DA, Reijneveld SA, Lanting CI, van Wouwe JP, Schuller AA (2019). Socio-economic inequality in oral health in childhood to young adulthood, despite full dental coverage. Eur J Oral Sci.

[9] National Institute of Dental and Craniofacial Research (2021). Oral health across the lifespan: working-age adults. Oral Health in America: Advances and Challenges.

[10] Andersen R, Newman JF (1973). Societal and individual determinants of medical care utilization in the United States. Milbank Mem Fund Q Health Soc.

[11] Armfield JM, Heaton LJ (2013). Management of fear and anxiety in the dental clinic: a review. Aust Dent J.

[12] Sermsuti-Anuwat N, Suwannimit R (2025). Influence of oral health literacy levels among foster caregivers on the use of dental services by foster children in Pak Kret, Thailand: a cross-sectional study. Spec Care Dentist.

[13] Villalobos-Rodelo JJ, Lucas-Rincón SE, Jimenez-Gayosso SI (2022). Characterizing socioeconomic inequalities in professionally applied topical fluoride treatment courses in schoolchildren from a developing country. J Immigr Minor Health.

[14] Cerón-Zamora E, Navarrete-Hernández JJ, Lara-Carrillo E (2020). Factors associated with the use of dental health services by Mexican schoolchildren to receive professionally applied topical fluoride. P R Health Sci J.

[15] Medina-Solís CE, García-Cortés JO, Robles-Minaya JL (2019). Clinical and non-clinical variables associated with preventive and curative dental service utilisation: a cross-sectional study among adolescents and young adults in Central Mexico. BMJ Open.

[16] Muñoz-Hernández O, Chertorivski-Woldenberg S, Cortés-Gallo G, Pérez-Cuevas R (2012). The Medical Insurance for a New Generation: a viable answer for the health needs of Mexican children. Salud Publica Mex.

[17] Ghaffari A, Graves KY, Bradbury RF, Harman JS (2025). Examination of rural-urban disparities in utilization of preventive dental procedures in the US pediatric population: a cross-sectional study. J Rural Health.

[18] Carrillo Ortiz AE, Olvera Fuentes CA, García Pérez A, Rodríguez Chávez JA, Villanueva Gutiérrez T, Flores Ruíz HM, Mora Navarrete KA (2024). Prevalence and severity of dental caries using ICDAS in predicting treatment needs in Mexican school-age children. J Clin Pediatr Dent.

[19] Jimenez-Gayosso SI, Robles-Bermeo NL, Scougall-Vilchis RJ (2024). High correlation of the decayed, missing, and filled teeth (DMFT) index with caries experience in first permanent molars: perspectives and implications in oral epidemiology from a cross-sectional study. Cureus.

[20] Villalobos Rodelo JJ, García Jau RA, Urias Barreras CM (2025). Factors associated with the development of dental caries among schoolchildren in northwest Mexico. J Clin Pediatr Dent.

[21] Pérez-Reyes Á, Becerra-Ruiz JS, Guzmán-Flores JM (2025). Influence of behavioral and sociodemographic factors on dental caries in Mexican children. Pediatr Rep.

[22] Márquez-Pérez K, Zúñiga-López CM, Torres-Rosas R, Argueta-Figueroa L (2023). [Reported prevalence of dental caries in Mexican children and teenagers]. Rev Med Inst Mex Seguro Soc.

[23] Moreno-Barrera A, Morales-Ruiz P, Ribas Pérez D, Flores-Fraile J, Castaño-Seiquer A (2023). Analysis and evaluation of dental caries in a Mexican population: a descriptive transversal study. Int J Environ Res Public Health.

[24] Arrieta-Vargas LM, Paredes-Solís S, Flores-Moreno M, Romero-Castro NS, Andersson N (2019). Prevalencia de caries y factores asociados: estudio transversal en estudiantes de preparatoria de Chilpancingo, Guerrero, México. Rev Odont Mex.

[25] Medina-Solís CE, Ávila-Burgos L, Borges-Yañez SA (2020). Ecological study on needs and cost of treatment for dental caries in schoolchildren aged 6, 12, and 15 years: data from a national survey in Mexico. Medicine (Baltimore).

[26] Casanova-Rosado JF, Casanova-Rosado AJ, Minaya-Sánchez M (2021). Self-reported dental caries by Mexican elementary and middle-school schoolchildren in the context of socioeconomic indicators: a national ecological study. Children (Basel).

[27] Winkler CH, Bjelopavlovic M, Lehmann KM, Petrowski K, Irmscher L, Berth H (2023). Impact of dental anxiety on dental care routine and oral-health-related quality of life in a German adult population-a cross-sectional study. J Clin Med.

[28] Dou L, Vanschaayk MM, Zhang Y, Fu X, Ji P, Yang D (2018). The prevalence of dental anxiety and its association with pain and other variables among adult patients with irreversible pulpitis. BMC Oral Health.

[29] Armfield JM, Stewart JF, Spencer AJ (2007). The vicious cycle of dental fear: exploring the interplay between oral health, service utilization and dental fear. BMC Oral Health.

[30] Mohammed Al-Dahan H, Ali Ismael S (2023). Early childhood caries: parents' knowledge, attitude and practice towards its prevention in refugee camps in Erbil, Iraq. BMC Oral Health.

[31] Aliakbari E, Gray-Burrows KA, Vinall-Collier KA, Edwebi S, Marshman Z, McEachan RR, Day PF (2021). Home-based toothbrushing interventions for parents of young children to reduce dental caries: a systematic review. Int J Paediatr Dent.

[32] Ruff RR, Niederman R (2018). School-based caries prevention, tooth decay, and the community environment. JDR Clin Trans Res.

[33] Ruff RR (2024). Caries incidence in school-based prevention programs in the presence of interval censoring. Children (Basel).

[34] Sharda S, Gupta A, Jyani G, Prinja S, Goyal A (2025). Modeling the cost-effectiveness of school-based supervised toothbrushing program in reducing the dental caries burden in India. Int J Paediatr Dent.

[35] Jimenez-Gayosso SI, Robles-Bermeo NL, Scougall-Vilchis RJ (2025). Care index, treatment needs, and bilateral occurrence of dental caries in the first permanent molars of Mexican schoolchildren. Cureus.

[36] Hernández-Martínez CT, Jiménez-Gayosso SI, Lucas-Rincón SE (2021). Dental pain prevalence associated with caries experience in pediatric patients in a clinical sample in Mexico. Braz Oral Res.

[37] World Health Organization (2013). Oral Health Surveys: Basic Methods. https://www.who.int/publications/i/item/9789241548649.

[38] Bagley SC, White H, Golomb BA (2001). Logistic regression in the medical literature: standards for use and reporting, with particular attention to one medical domain. J Clin Epidemiol.

[39] de Diputados del HC (2014). Reglamento de la Ley General De Salud En Materia De Investigacion Para La Salud. https://www.diputados.gob.mx/LeyesBiblio/regley/Reg_LGS_MIS.pdf.

[40] Mazzoleni S, Zuccon A, De Matteo S (2024). Correlation of parental and child dental plaque levels: a clinical study. Appl Sci.

[41] National Institute of Dental and Craniofacial Research (2021). Oral health across the lifespan: children. Oral Health in America: Advances and Challenges.

[42] Nagdev P, Iyer MR, Naik S (2023). Andersen health care utilization model: a survey on factors affecting the utilization of dental health services among school children. PLoS One.

[43] Zaborskis A, Kavaliauskienė A, Levi S, Tesler R, Dimitrova E (2023). Adolescent toothbrushing and its association with sociodemographic factors-time trends from 1994 to 2018 in twenty countries. Healthcare (Basel).

[44] Hedges I, Flynn B, Vujicic M, Smith A, Ward L (2025). American Dental Association. Improving dental care access for vulnerable populations. July.

[45] Curtis B, Evans RW, Sbaraini A, Schwarz E (2007). Geographic location and indirect costs as a barrier to dental treatment: a patient perspective. Aust Dent J.

